# Frequency of OBI among Patients with Autoimmune Hepatitis

**DOI:** 10.31557/APJCP.2020.21.9.2555

**Published:** 2020-09

**Authors:** Golnaz Hayatdavoudi, Manoochehr Makvandi, Ali Timori, Alireza Samarbafzade, Somayeh Biparva Haghighi, Akbar Bavi, Pezhman Alavinejad, Hossein Keyvani

**Affiliations:** 1 *Infectious and Tropical Diseases Research Center Health Research Institute, Ahvaz Jundishapur University of Medical Sciences, Ahvaz, Iran. *; 2 *Virology Department, School of Medicine, Ahvaz Jundishapur University of Medical Sciences, Ahvaz, Iran. *; 3 *Faculty of Medicine, Ahvaz Jundishapur University of Medical Sciences, Ahvaz, Iran. *; 4 *Division of Gastroenterology and Hepatology, Imam Khomeini hospital, Ahvaz Jundishapur University of Medical Sciences, Ahvaz, Iran. *; 5 *Virology Department, School of Medicine, Iran University of Medical Sciences, Tehran, Iran. *

**Keywords:** Autoimmune hepatitis, anti-HBc, nested Polymerase Chain Reaction, real, time Polymerase Chain Reaction

## Abstract

Autoimmune hepatitis (AIH) is recognized as a serious disease in which the body’s immune system attacks liver cells so untreated patients may consequently suffer from liver cirrhosis, hepatocellular carcinoma (HCC) and liver failure. The role of viral infection may be involved in AIH. Presence of anti-HBc alone is a predictive signal of potential OBI. Thus, this study was conducted to evaluate the rate OBI among the patients with AIH.

**Methods:**

The sera of 20 consecutive patients with AIH were collected and tested for LFT (ALT, AST, ALP elevation), Immunoglobulin (IgG) level, bilirubin, anti -LKM-1, ASMA, ANA in titer, HBsAg, HBcIgG. The patients’ sera were also tested for HBV DNA by nested PCR and Real-time PCR.

**Results:**

Out of 20 patients, 10 (50%) were males and 10 (50%) females. The patients’ ages ranged from 25 to 71 years with the mean age of 44.5±13.4. All patients’ had elevated abnormal ALT and AST but their level of alkaline phosphatase was normal among the patients. All patients had IgG level>1.5 times upper than the normal limit. The patients’ sera were negative for HBsAg and HBV DNA (by nested PCR and real- time PCR). Only 2 (10%) females with AHI type 1 (positive ANA, ASMA in titers >1:100 were positive for HBcIgG while no OBI detection was found among the males (p=0.005)). All diagnosis of the AHI was confirmed by pathologist. The level of ALT, AST among the cases with positive and negative OBI were (p=0.000) and (p=0.003), respectively.

**Conclusion:**

In the present study, two OBI female patients with type 1 AIH were positive for anti-HBc but negative for HBsAg and HBV DNA. With regard to the consequences of OBI, prior to prophylactic treatment, it is recommended to screen HBV markers including anti-HBc in all diagnosed patients with AIH.

## Introduction

Autoimmune hepatitis (AIH) is a chronic status that may develop to cirrhosis, Hepatocellular Carcinoma, HCC and liver failure (Sahebjam, 2015; Krawitt, 2006). The exact cause of autoimmune hepatitis remains unknown but several factors including genetic and environmental influences and viral infections may trigger the disease (Liberal et al., 2014; Liberal et al., 2013; Rigopoulou et al., 2013; Sezaki et al., 2003). So far, two types of AIH have been established: type 1 (AIH-1) is characterized by the presence of antinuclear antibodies (ANA) and anti-smooth muscle antibodies (anti-SMA) and type 2 (AIH-2) is defined by the positivity for anti-liver kidney microsomal type 1 antibodies (anti-LKM-1) or presence of anti-liver cytosol type 1 antibody (anti-LC-1) (Liberal et al., 2013). It has been reported that viral infection can interrupt immune self-tolerance and cause an autoimmune process that may result in the development of autoimmune diseases (Rigopoulou et al., 2013; Sezaki et al., 2003; Nicole et al., 2016).

Moreover, patients with AIH may have occult hepatitis B infection (OBI) and also immunosuppressive therapy can cause the reactivation of OBI, which makes the diagnosis and treatment more difficult for physicians (Gatselis et al., 2015; Zachou et al., 2013). HBV infection is a major cause of chronic liver disease, affecting more than 240 million people worldwide (Rosa et al., 2017). HBV is the tenth leading cause of death in the world and infections result in 500,000 to 1.2 million deaths annually due to chronic hepatitis, cirrhosis and hepatocellular carcinoma (HCC) (Thu et al., 2019). Iran has a HBV endemicity (around 2-7%), (Smolle et al., 2012; Zidan et al., 2012). HBV infection accounts for acute and chronic hepatitis and inactive carrier phase to OBI (Makvandi, 2016). Two classes of OBI have been defined: first, seropositive OBI defined as the presence of HBV DNA (<200 IU/mL) in serum/liver by sensitive diagnostic tests such as Real- Time PCR; second is seronegative OBI described as the absence of anti-HBc or anti-HBs in the serum while HBV DNA is detectable in the liver cells and Peripheral Blood Cells (PBMC) (Makvandi, 2016; Doda et al., 2014). The transmission of OBI possibly leads to an increased risk of the disease progression (Candotti et al., 2017) 

It is well-known that anti-HBc alone is a predictive signal of potential OBI (Roman, 2018). OBI has been associated with extrahepatic manifestations including rheumatoid arthritis, B-cell non-Hodgkin lymphoma, membranous glomerulonephritis hepatocellular carcinoma (HCC) and autoimmune hepatitis (Sinha et al., 2016; Xiong et al., 2015; Muto et al., 2018). 

Autoimmune hepatitis (AIH) is a progressive chronic hepatitis which affects all ages and both sexes but is more common among females (Dong et al., 2018). Although the causes of autoimmune hepatitis (AIH) disease remain unknown, genetic and environmental factors and viral hepatitis B and C may provoke the disease (Vento et al., 2004; Hui et al., 2006). The occurrence of mutations in the ‘a’ determinant amino acid of the S gene at positions 124-147aa was found to be a major contributing factor to OBI (Zhu et al., 2016). 

Moreover, detection of OBI is very crucial in patients receiving systemic chemo-radiation or immunosuppressive therapy as the risk of OBI reactivation may result in fulminant hepatic failure (Makvandi, 2016). Therefore, this study aimed to investigate the prevalence of OBI in AIH patients in Ahvaz city, Iran. Ahvaz is the capital of Khuzestan province located in southwest region of Iran. 

## Materials and Methods

In this cross-sectional study, 119 patients with autoimmune hepatitis referred to gastroenterology clinics of Imam Khomeini Hospital in Ahvaz. They were selected during April 2015 to April 2016. The paraclinical tests, including serum levels of alanine aminotransferase (ALT), aspartate aminotransferase (AST), alkaline phosphatase (ALP), serum bilirubin level, and serum IgG level and autoantibody tests, including immunofluorescence tests for ANA (antinuclear antibody) and ASMA (Anti-smooth muscle antibody and anti LKM-1 (liver kidney microsomal antibody type-1) were extracted from their medical files. In addition, anti LKM-1 and ASMA titer exceeding 1:100 and ANA ≥1:100 were considered as positive according to the manufacturer guideline. Exclusion criteria were considered as follows: patients with Wilson’s disease, alpha-1 antitrypsin deficiency, fatty liver, patients using Atorvastatin, Isoniazid, Diclofenac, Propylthiouracil, and Infliximab, primary biliary cirrhosis (PBC) and primary sclerosing cholangitis (PSC). Finally, 20 patients including 10 males and 10 females met AHI criteria and were registered in this survey. The patients’ ages were between 25 to 71 years with the median age 40.5. All the patients had clinical history of jaundice, dark urine, vomiting, abdominal pain, nausea, malaise, anorexia, pale stool, melena, weight loss, chronic diarrhea, fatigue. The patients’ sera were tested for HBV serological markers including HBsAg, HBcIgG, antibody by ELISA test (Diaper Co., Italy) according to manufacturer’s instructions. The liver biopsies were carried out for each patient to confirm the AIH disease.

*DNA Extraction*

Viral nucleic acid was extracted from 200 μL of each patient’s serum using the high pure viral nucleic acid kit (Roche Applied Science, Germany) according to the manufacturer’s procedure.

*Nested Polymerase Chain Reaction*

The nested PCR for detection of “S” region of HBV genome was performed for all the tissue samples (Sitnik et al., 2004).

For the first round, the outer primer forward FHBS1 5’-GAGTCTAGACTCGTG GTGGACTTC-3’ and reverse primer RHBS1,5’-CGTGGTGGACTTCTCTCAATTTTC-3’ were used. The PCR reaction mixture containing 5 µL of the extracted DNA from each sample, 0.5µL dNTP (10 Mm), 2.5 µL PCR buffer (10x), 0.15 μL 5U Taq DNA polymerase (Roch, Germany), 50 pmol/µL of the FHBS1 and RHBS1 primers, 0.5 µL dNTP (10 Mm) and 15.85 µ L distilled water up to 25 μL. The samples were placed in the thermal cycler (Techne Company, UK) and amplification was carried out with initial denaturation at 94°C for five minutes, followed by 30 cycles at 94°C for 30 seconds, annealing at 56°C for 30 seconds and extension at 72°C for 30 seconds. For the second round, the inner forward primer FHBS2, 5’-AAATKGCACTAGTAAACTGAGCCA-3’ and reverse primer RHBS2, 5’-GCCARGAGAAAC GGRCTGAGGCCC-3’ were used. Five µL of the PCR product was added to reaction mixture containing the same components mentioned in the first run including dNTP, PCR buffer and Taq DNA polymerase with 50 pmol/µL of each of FHBS2 and RHBS2 primers. Amplification was carried out in the thermal cycler with the same program as the first round. The nested PCR product (417 bp) was analyzed by 2% agarose gel electrophoresis.

*Quantitative Real-Time PCR*

The extracted DNA sera of all AIH patients including positive for HBc antibody were quantified for HBV DNA by Real-Time PCR kit (Cobas TaqMan HBV test, Roche; cutoff 6 IU/ml equivalent to 35 copies/ml) according to manufacturer instruction. 

*Ethical approval*

This project with ethic code IR.AJUMS.REC.1394.152 was approved by the ethic committee of the deputy of research and affairs of the Faculty of Medicine Ahvaz Jundishapur University of Medical Sciences, Ahvaz Iran. All experiments were performed in compliance with relevant laws and institutional guidelines and in accordance with the ethical standards of the Declaration of Helsinki. Written ethnic consent was obtained from each individual participated in this research work. 

*Statistical analysis*

Statistical analyses were performed via SPSS software (Statistical Product and Service Solutions, version 22.0). Qualitative variables differences were evaluated using the chi-square or the Fisher exact test. Quantitative variables were compared using the Student’s t-test or Mann-Whitney U test as appropriate. All p values < 0.05 were considered significance.

## Results

All the patients’ sera elevated more than five times higher than the normal limit of ALT(median (range) 198 IU/L), AST (median range: 196 IU/L), all the patients showed normal level of alkaline phosphatase (ALP) (median range: 188 IU/L). All the patients raised IgG level ≥1.5 times upper than the normal limit. Nineteen (95%) patients including 10 females and 9 males showed positive AHI type 1 (positive ANA, ASMA in titers >1:100) and one male (5%) was positive for AHI 2 (positive ANA, Anti-LKM antibody type-1). All diagnoses of the AHI were approved by a pathologist. The elevation of gamma globulin levels along with elevation of SGPT and SGOT are important markers of AIH disease activity. The sera of all patients were negative for HBsAg (ELISA) and HBV DNA according to nested and real-time PCR analyses. The sera of two (10%) females with AHI type 1 were positive for HBcIgG, indicating the presence of OBI among them. No OBI was detected among the males (p=0.000). Table 1 summarizes the distribution of AHI and OBI among the participants’ different age groups.

**Table 1:** Distribution of OBI

Characteristics	Total	OBI (+) n=2	OBI (--)18	P value
Sex (F/M)	20	2/10	0/10	0.47
Age(years) median age	40.2	40 ±13	41 ±4	1.00
<45	8	0	8	0.49
>45	12	2	10	
ALT (2-35 U/L) n=20 Median (range)	198	314(302-326)	199.6(80-845)	0.000
AST (2-35 U/L) n=20 Median (range)	196	212(206-2018)	198(75-812)	0.003
ALP (98-279 IU/L) Median (range) n=20	188	220(200-240)	219(218-220)	1.00
Anti-HBc	20	2	18	0.05
ANA ≥ 1:100	20	2	18	0.48
ASMA ≥1;100	20	2	18	0.48
LKM-1≥ 1: 100	20	0	1	
IgG, mg/ dI (656-1351) Median range	20	2(2200)	18(2250)	0.35

Alanine aminotransferase (ALT); Aspartate aminotransferase (AST); Alkaline phosphatase (ALP); ANA (antinuclear antibody); ASMA Anti-smooth muscle antibodies and anti LKM (liver kidney microsomal antibody).

## Discussion

Autoimmune hepatitis is considered as a chronic liver inflammation and a great public health problem that occurs worldwide with unknown etiology. Untreated autoimmune hepatitis can result the liver cirrhosis, hepatocellular carcinoma and finally, liver failure. AIH commonly occurs in young, adolescence or early adulthood. The exact prevalence of AIH and its association with viral infection is very limited and remain unknown (1, 2, 3, 4).

In the presence study, all the patients had raised SGPT(224 ±70) and SGOT(198 ± 18) level which were about 6 times upper than the normal limit. The level of alkaline phosphatase elevation was in the normal range (174±14). The fact that of SGPT and SGOT raised up to 3-10 times more than the normal level and the normal alkaline phosphatase level can be a good indicator for tracking AHI (Alvarez et al., 1999). The abnormal rising of alkaline phosphatase level has been found in diseases such as autoimmune Hepatitis-Primary Biliary Cirrhosis, primary sclerosing cholangitis (PSC) (Olsson et al., 2009). Moreover, drug-induced autoimmune-like hepatitis (DIAH) such as propylthiouracil, minocycline and nitrofurantoin have been reported (Czaja, 2011; Angelberger et al., 2011).

In the current research, 19/20 (95%) patients were positive for ANA and ASMA. This indicates that the presence of AIH type 1 is dominant among patients with AIH in this region. On the other hand, one (5%) male patient was positive for ANA and anti -LKM-1; this condition reveals the presence of AIH type 2. Previous studies indicated that the AIH type 1 is common in north America and Europe, Asia while AIH type 2 is predominantly found in children, About less than 10% of AIH are type 2 were reported in north America and Europe (van Gerven et al.,2016; Kaur et al., 2014). 

The prevalence of HLA-DRB1 has been reported among patients with AIH types 1 and 2 in different regions of the world (Baharlou et al., 2016). In the present study, the frequencies of HLA-DRB1(*)03, HLA-DRB1(*)04, HLA-DRB1(*)08 among the patients with AIH were not evaluated. In our survey, all the patients with AIH have raised gamma globulin level with elevation of SGPT and SGOT levels. The elevation of gamma globulin levels along with elevation of SGPT and SGOT are important markers of AIH disease activity (Alvarez et al., 1999). 

So far, it has been described that women are more prone to AIH than male. The present study documented equal rate of AIH in women (50%) and men (50%). 

Recently, an association of occult hepatitis B infection (OBI) rheumatoid arthritis has been reported among patients autoimmune hepatitis (Chen et al., 2019). 

At present, the golden standard test for OBI diagnosis is the detection of HBV DNA in liver by real-time PCR. In some cases of seropositive OBI, the HBcIgG is detected while HBV DNA is undetectable in the patients’ sera. In fact, HBV DNA is detectable in the liver biopsies even < 200 IU/ml has been reported by the quantitative real-time PCR. In this survey, all the patients were negative for HBsAg; only 10% of female patients were positive for HBc IgG but negative for HBV DNA by nested PCR and real-time PCR. This actually indicates the presence of OBI among these patients. To confirm OBI, the detection of HBV DNA in the liver biopsies is required by the real-time PCR which was not implemented in this study. Now, it is known that anti-HBc alone is a predictive signal of potential OBI (Roman, 2018 )

Both the OBI patients were type 1 AIH with elevation of ALT and AST which were 6 times upper than normal level and Gamma globulins (IgG) levels were ≥1.5 times upper than normal limit while their Alkaline phosphatase level was normal. 

Georgiadou et al., (2009) reported that out of 196 patients with the autoimmune liver disease, 66 individuals were diagnosed for AIH. Among them, 9 (% 13.6) patients were positive for OBI.

In the present study, 90% of the patients with AIH were negative for HBV markers. The association of other viruses such as hepatitis C virus (HCV), hepatitis B virus (HBV), cytomegalovirus (CMV), hepatitis A virus (HAV), Epstein-Barr virus (EBV), human herpesvirus 6, hepatitis E virus and measles have been evaluated in patients with AIH (Christen et al., 2018; Taubert et al., 2018). The association of the aforementioned viruses with AIH was not studied among the patients. 

In conclusion, as the results revealed, two OBI female patients with type 1 AIH were positive for anti-HBc but negative for HBsAg and HBV DNA by nested-PCR and Real-time PCR. This indicated the association of OBI with AIH. It is currently known that anti-HBc alone is a predictive signal of potential OBI. Therefore, considering the subsequent consequences and to manage and treat OBI, it is recommended to screen all diagnosed patients with AIH for HBV markers including anti-HBc prior to prophylactic treatment. 
